# Evaluation of a Tangential Map-Based Nomogram for Intrastromal Corneal Ring Segments' Implantation in Keratoconus: One Year Results

**DOI:** 10.1155/2020/3983508

**Published:** 2020-02-19

**Authors:** Adel Galal Zaky, Mahmoud Tawfik KhalafAllah, Abdelrahman Elsebaey Sarhan, Moataz Faiz Elsawy

**Affiliations:** Menoufia Faculty of Medicine, Shebeen El-Kom, Egypt

## Abstract

**Purpose:**

To evaluate a new tangential map-based nomogram versus the axial map-based nomogram for ICRS in keratoconus.

**Methods:**

A prospective case series study including 64 eyes of 64 patients who underwent ICRS implantation. Cone location was determined for each eye with two maps: the axial and the tangential. Appropriate ring selection was determined using two surgical nomograms: axial map-based and tangential map-based. Visual, refractive, and topographic outcomes were assessed before, as well as at 3, 6, and 12 months after ICRS implantation.

**Results:**

The cone location, and consequently the ring selection, was significantly different in the two nomograms with a “centralization tendency” in the tangential map. In the axial group, UDVA and CDVA improved from 0.12 ± 0.04 and 0.24 ± 0.08 to 0.28 ± 0.08 and 0.4 ± 0.1, respectively. Similarly, MRSE substantially decreased from −6.7 ± 3.3 to −1.2 ± 1.1*D* at 12 months after the procedure. In the tangential group, UDVA and CDVA improved from 0.09 ± 0.06 and 0.2 ± 0.1 to 0.5 ± 0.2 and 0.7 ± 0.2, respectively. MRSE substantially decreased from −4.9 ± 1*D* at 12 months after the procedure. In the tangential group, UDVA and CDVA improved from 0.09 ± 0.06 and 0.2 ± 0.1 to 0.5 ± 0.2 and 0.7 ± 0.2, respectively. MRSE substantially decreased from −4.9 ± 1*D* at 12 months after the procedure. In the tangential group, UDVA and CDVA improved from 0.09 ± 0.06 and 0.2 ± 0.1 to 0.5 ± 0.2 and 0.7 ± 0.2, respectively. MRSE substantially decreased from −4.9 ± 1*P*=0.01^*∗*^. Similarly, the gain in the CDVA was 0.4 and 0.15 in the tangential and axial groups, respectively, at 12 months, *P*=0.01^*∗*^. Similarly, the gain in the CDVA was 0.4 and 0.15 in the tangential and axial groups, respectively, at 12 months,

**Conclusion:**

The tangential map-based nomogram attained better visual and refractive outcomes at 1 year. In addition, the cone location was significantly different between both maps with a centralization tendency in the tangential one.

## 1. Introduction

Keratoconus (KC) is a bilateral, progressive, noninflammatory disease of the cornea which often leads to high myopia and astigmatism which impair the acuity and quality of vision. It has an estimated prevalence of approximately 1 in 2000 and an incidence between 50 and 230 per 100,000 [1, 2]. In its early stages, KC can be managed conservatively via spectacles or rigid contact lenses. In its advanced stages, deep lamellar keratoplasty and penetrating keratoplasty (PK) are considered. However, potential complications and technical challenges raised the need for an alternative [3, 4].

Intrastromal corneal ring segments (ICRS) enriched the armamentarium for KC, with promising results in topographic regularity and uncorrected distant visual acuity (UDVA), raising the hopes to reduce the need for keratoplasty [5, 6]. ICRS vary in their design, arc length, thickness, inner diameter, and its orientation in implantation. Appropriate ring selection depends on the cone location and/or asymmetry, spherical error, astigmatism, *Q* value, and corneal topography; however, no nomogram is agreed upon [7]. All of these parameters are measured objectively with standard techniques. Cone location, in contrast, is neither agreed for its definition nor its assessment method, including the tomographic map to be used.

Even the current manufacturers as well as many surgeons depend on the axial map as a guide for the cone location and subsequently the appropriate ring selection and implantation. The tangential map, however, was superior in many aspects, including detection of subclinical KC and contact lens fitting in KC [8, 9]. Given the prior advantage, surgical nomograms should have considered the tangential map for cone location.

In this study, we aimed to compare the axial map-based versus a tangential map-based nomogram for ICRS implantation in keratoconus. The comparison was planned in terms of preoperative cone location matching, in addition to postoperative visual, refractive, and topographic outcomes.

## 2. Patients and Methods

This was a prospective case series study conducted at Tiba Eye Center, Shebin Elkom, Menoufia, Egypt, during the period from April 2017 to July 2018 on patients with KC who are candidates for ICRS and meet the eligibility criteria. All study procedures were approved by the Ethical Committee of Menoufia Faculty of Medicine and were in accordance with the declaration of Helsinki.

We included patients with clinical keratoconus, diagnosed with pentacam (WaveLight® Allegretto Oculyzer Erlangen, Germany), with clear central cornea, age between 21 and 49 years, minimum corneal thickness of 400 *μ*m at the optical implantation zone, mean keratometry between 45 and 60*D*, contact lens intolerance, an uncorrected distant visual acuity (UDVA) not better than 0.5 decimal, and no visual dysfunctions other than keratoconus. Pregnant or breast-feeding mothers and patients with other ocular or systemic pathologies were excluded.

The sample size was calculated using Power Analysis Sample Size software (version 15, NCSS, LLC) setting the type 1 error (a) at 0.05 (95% confidence interval (CI)) and the power (1-b) at 0.8. The sample size was calculated to detect a difference of at least 0.25*D* in sphere and cylinder between before surgery and after surgery (effect size). The result of the sample size calculation was 25 eyes per group.

**Figure 1:**
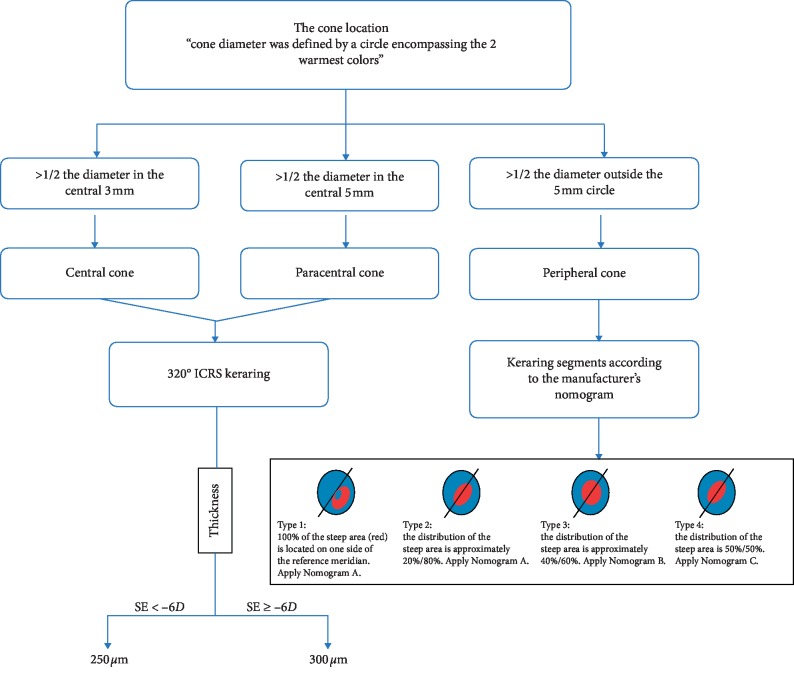
The surgical nomogram applied for corneal ring selection. For peripheral cones, ring segments were implanted according to nomograms A, B, or C (see Supplementary materials ([Supplementary-material supplementary-material-1])).

The cone location was labeled as central, paracentral, or peripheral according to the extent of the cone area. A circle that encompasses the 2 warmest colors was delineated, and its diameter was measured. The cone was central or paracentral if more than half of the diameter fell within the central 3 or 5 mm, respectively. Otherwise, it was labeled as a peripheral one. The cone location was determined for each case with both axial and tangential maps and compared for matching. For each one, the same surgical nomogram was applied respecting the cone location as determined by the map used. For central and paracentral keratoconus, 320° ICRS Keraring (Mediphacos Ltd., Minas Gerais, Brazil) was applied with the thickness of 300 and 250 *μ*m for SE of more than −6.00*D* and less than −6.00*D*, respectively. For peripheral cones, Keraring segments were implanted according to the manufacturer's nomogram tables as shown in Figure 1.

An alternative scenario analysis was planned. This is to determine the cone asymmetry classification type, as reported in Keraring manufacturer's nomogram, by both maps. Cone asymmetry classification was determined for each eye as proposed by the manufacturer in relation to the reference meridian (on one side, 20 : 80, 40 : 60, and 50 : 50) using the axial map and then matched to that using the tangential map. Two independent authors visually assessed the cone asymmetry in both maps and discrepancies were resolved by discussion or by the senior author. After that, matching the cone pattern and the subsequent nomogram (*A*, *B*, or *C*) was conducted to emphasize the discrepancy, if any, between both maps as a guide for ICRS implantation.

All patients were carefully examined preoperatively. Preoperative evaluation comprised uncorrected (UDVA) and corrected distance visual acuity (CDVA) in decimal units, manifest and cycloplegic refraction, slit lamp examination including applanation tonometry, fundus examination, and corneal topography with a rotating Scheimpflug device (WaveLight® Allegretto Oculyzer, Erlangen, Germany). The primary outcome was CDVA at 12 months after the operation. Secondary outcomes were UDVA, CDVA, manifest refraction SE (MRSE), *K*_max_, *K*_m_, and *Q* value at 3, 6, and 12 months. The outcome assessors were blinded regarding the type of map guide used.

Aiming at a standard procedure, ICRS implantation was carried out by the same surgeon (A. S) under local anesthesia using a femtosecond laser (VisuMax, Carl Zeiss Meditec AG, Inc., Jena Germany). The stromal tunnel was created at approximately 70% of corneal depth. The pockets were opened using a blunt sinskey, and the rings were implanted. Soft bandage contact lens was placed immediately at the end of surgery in all cases, and patients were instructed to avoid eye rubbing. All patients received the same postoperative treatment of topical antibiotic and steroid (Fortymox® q.i.d and Predforte® q.i.d).

Data analysis was conducted using SPSS v.24 software (SPSS Inc., Chicago, IL, USA). Continuous variables are presented as mean ± SD. Normal distribution of the data was checked by the Kolmogorov–Smirnov test. Student's *t*-test was used to compare the means of the outcomes between both groups. The paired *t*-test was used to compare preoperative and postoperative values of UDVA, CDVA, SE, *K*_max_, *K*_mean_, and *Q* value. The mean change in the above variables was analyzed using Student's *t*-test. Chi-squared test was used to compare categorical variables. The *P* value <0.05 was considered statistically significant in all these tests.

## 3. Results

Sixty-four eyes of 64 patients, 36 males and 28 females, were included in the study. They were divided into two groups according to the nomogram applied, and in other words, the map used to locate the cone: group *A* (Axial group) included 36 eyes, of which 24 were males and 12 females, while group *B* (Tangential group) included 28 eyes, of which 12 were males and 16 females. Mean age in both groups were 27.6 ± 3.8 and 23.7 ± 3.5 years for the axial and the tangential groups, respectively.  Table 1 plots the baseline characteristics for both groups, showing no significant differences, ensuring baseline homogeneity of the study population. For both groups, no intra- or postoperative complications were detected.

For the cone location, labelling the axial map as the reference one, the matching rate between both maps was 67.6%, 62.5%, and 38.1% for central, paracentral, and peripheral cones, respectively, which was statistically significant (*P*=0.01). Moreover, the peripheral group showed the greatest discrepancy where 26 out of 42 (61.9%) were labeled as paracentral on the tangential map although displayed as peripheral on the axial map.  Table 2 plots the matching rate between both maps, while Table 3 plots different types of ICRS implanted in either group accordingly with a significant difference in the ring implanted (*P*=0.01^*∗*^).

**Figure 2:**
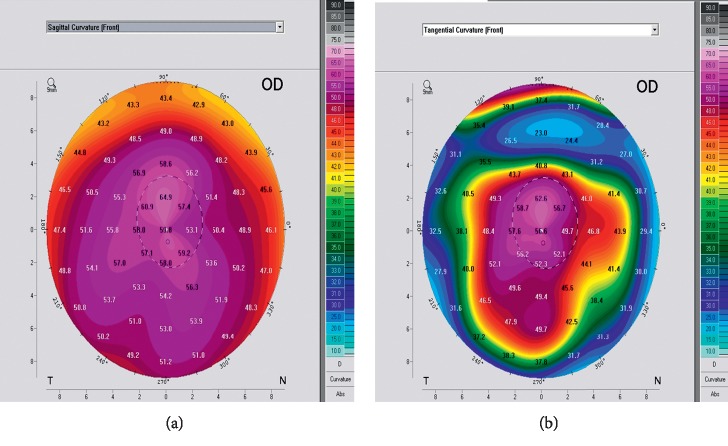
Pentacam for the same eye: axial map (a) shows peripheral cone and tangential map (b) shows paracentral cone.

**Figure 3:**
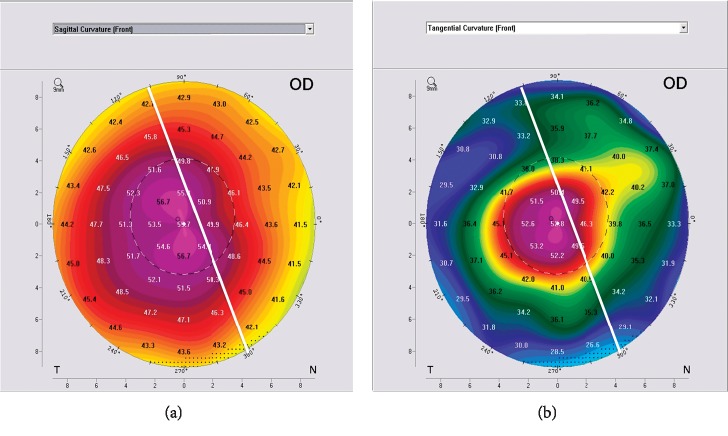
Pattern asymmetry classification showing 20 : 80 cone pattern in axial and tangential map.

**Figure 4:**
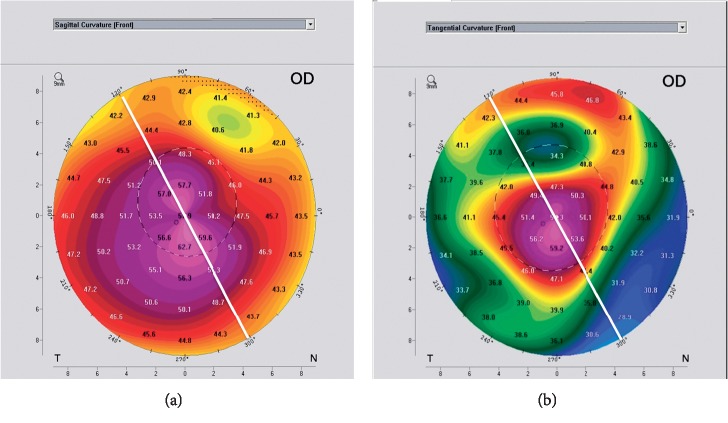
Pattern asymmetry classification showing 40 : 60 and 50 : 50 cone pattern in axial and tangential maps, respectively.

For the cone symmetry classification proposed by Kera manufacturer's nomogram (Figure 1), a significant difference was detected (*P*=0.01^*∗*^) as shown in Table 4. For cone asymmetry classification, the highest matching rate between both maps was lower than that for cone location (25% versus 67.6%), potentiating the evidence for the discrepancy between both maps. Figures 2–4 plot the process of visual judgment for both situations.

In both groups, UDVA and CDVA significantly improved at 3, 6, and 12 months after the surgery. Similarly, a significant reduction was noted in MRSE, *K*_max_, and *K*_m_ in both groups at 3, 6, and 12 months postoperatively. As well, the Q value significantly changed from −1.28 preoperatively to −0.4 at 12 months (*P*=0.001) and from −1.38 preoperatively to −0.38 ± 0.5 at 12 months (*P*=0.003) in the axial and the tangential groups, respectively. In addition, a significant increase in the thinnest location thickness was documented for both groups at all follow-up visits. Table 4 plots in detail the postoperative outcomes for either group at all follow-up visits. No significant correlation could be detected between the *K*_m_ or *K*_max_ with any of the visual or refractive outcomes.

The value of change from the baseline was calculated and compared between both groups. At 3 months, the tangential group showed significantly better gain in UDVA (0.2 ± 0.09) versus (0.11 ± 0.07) in the axial group (*P*=0.02). In addition, at 6 and 12 months, the tangential group showed significantly better gain in UDVA (0.3 and 0.35 versus 0.13 and 0.15 in the axial group at 6 and 12 months, respectively) and CDVA (0.25 and 0.4 versus 0.13 and 0.15 in the axial group at 6 and 12 months, respectively). MRSE reduction was also higher in the tangential group (5.4 and 5.4*D* versus 3.7 and 3.9*D* in the axial group at 6 and 12 months, respectively). Tables 5 and 6 show the postoperative outcome and the value of change at different time points for both groups.  Table 7 plots the different outcomes at all time points for cases with discrepancy in the cone location between both maps. In addition, the 12-month change values are compared between both groups for different cone locations as shown in Table 8.

## 4. Discussion

Intrastromal corneal ring segments (ICRS) have been proposed and investigated as an additive surgical procedure for keratoconus correction [10] aiming at visual improvement and delaying corneal graft in patients with keratoconus [11]. Nonetheless, surgical nomograms are extremely variable, relying mostly on surgeons' experience rather than validated studies, yielding variable unpredictable outcomes. Moreover, manufacturers' nomograms have not been validated in an unusual practice [12, 13].

Keraring proposed its nomogram in 2009, relying on cone asymmetry in relation to the reference meridian. As well, many other designs were proposed and widely used: 355°, 340°, and 320°. Most surgical nomograms rely upon the axial map to determine the cone location or asymmetry. Instead of constructing a validated and reliable nomogram, simple unexplained instructions are provided with each ring [14–16]. Few attempts were reported to create validated nomograms or simulated models to avoid unpredictable events [17]. However, the issue was not resolved.

Axial and tangential maps do differ in many aspects, and a lot of studies affirm this. In apical corneal power, which is sensitive to minor changes in radius, the tangential map was superior to the axial one in local radius of curvature measurement, consequently, superior in apical power assessment. That is why tangential map was recommended for apical clearance method of contact lens (CL) fitting in keratoconus [18]. The tangential map could consistently show greater apical power in all grades of keratoconus [19]. In contact lens fittings, it was reported that tangential map is of paramount importance to the axial map in terms of cone shape and location accuracy as well as peripheral corneal changes delineation [20]. Moreover, the tangential map was superior to the axial map in screening for subclinical keratoconus [8, 21, 22].

The impact of the cone location on the outcomes after corneal cross-linking and corneal rings is a matter of debate. In 2012, Greenstein investigated how preoperative cone location can predict and/or influence the 1-year outcomes of CXL in progressive keratoconus and post-LASIK ectasia. In keratoconus, obtained flattening effects changed with different cone locations. Maximum *K* was flattened by a mean of 2.7*D* and 0.9*D* in the central and peripheral cone groups, respectively. This was attributed to a nonhomogenous effect of CXL (central versus peripheral effects) and inherent corneal biomechanics. Both factors could be encountered with ICRS [23].

In the present study, 64 eyes of 64 patients were included. We aimed to evaluate if the cone location substantially differs between the axial and tangential maps. In addition, we aimed to investigate potential effects of any discrepancy on visual and topographic outcomes after ICRS implantation. While we assessed the visual and topographic outcomes at 3, 6, and 12 months postoperatively, we will emphasize the 1-year outcomes in the discussion section.

In our study, the mean age was 27.6 and 23.7 years in the axial and the tangential groups, respectively. While there was no statistically significant difference between both groups, the age may seem relatively high compared to the keratoconic population. The effects of aging on keratoconic corneas are the areas of debate. For normal population, corneal stiffness increases with age due to changes in the stromal collagen. This led to the assumption that the same could happen with KC which can stabilize or, at least in part, slow the disease progression [24]. Millodot et al. explored how central corneal curvature and CDVA changed after at least twenty years of KC in 67 patients, the longest follow-up period authors could find in the literature. Interestingly, the central curvature along with CDVA showed progressive changes for 28 years after the onset of the disease before stability predominates [25].

To extend the latter point, the potential correlation between ICRS outcomes and age was explored. An outstanding hurdle in many ICRS studies, including ours, is the narrow age ranges included. Torquetti et al.'s study was among the early studies to report regression of ICRS outcomes in progressive keratoconus in young patients [26, 27]. Refuting this, Cueto et al. reported stability of the obtained outcomes by Ferrara rings in patients above or below thirty-years age [28]. Vega-Estrada et al. reported a five-year follow-up for ICRS for patients' age ranging from 15 to 56 years. There was no correlation between the age, and they obtained changes in the mean *k* readings. Nonetheless, the retrospective nature of the study along with the different techniques (femto-assisted and mechanical), and the nomograms applied may question the reliability of this correlation. One more point to highlight is restricting the inclusion to those with stable refraction before the procedure, an additional limitation for the obtained correlation [29].

The cone location, as previously defined, was matched only in 67.6%, 62.5%, and 38.1% for central, paracentral, and peripheral locations, respectively. Such discrepancy in matching cone locations might be attributed to the validity of the cone definition applied rather than a true difference between both maps. To compensate for this, we planned for an alternative scenario analysis. Given the fact that the manufacturer's nomograms were the most widely applied, what would have been the case if the tangential map replaced the axial one in these nomograms? The cone asymmetry classification proposed by the manufacturer is a visual interpretation of the warm areas on the map. The matching rate between both maps was highest in nomogram C, the 50 : 50 cones. Nonetheless, this “highest” matching rate was 25%, a considerably low rate, affirming inherent differences between both maps.

It is not clear, after two decades of practice, how the cone location impacts, if any, the ICRS outcomes. Scanning manufacturers' nomograms reveal how centrality and symmetry of the cone could alter the ring choice; however, there was no evidence to support. To get over this, some studies restricted their inclusion to only one cone location. Cueto et al. reported the outcomes of one or two Ferrara ring segments in central KC with regular astigmatism [30], while Lisa et al. implanted the 210° Ferrara ring in central KC [31]. In the latter two studies, the same surgeon carried out the procedure, and the inclusion was limited only to stages I and II according to the Amsler–Krumeich classifications. However, the value of change in the reported outcomes was different which may indicate that long-arc rings may achieve better endpoints.

Featuring the crucial role of cone location, some other studies evaluated the outcomes in paracentral KC. A longitudinal follow-up study reported significantly improved UDVA and CDVA 6 months after Ferrara ring implantation which remained stable for five years [28, 32]. In contrast, a recent study stated a clear conclusion that cone location was not related neither to the visual nor the topographic outcomes [33]. However, the small sample size, 19 eyes, along with the short-term follow-up (6 months), and the retrospective nature of the study may question the conclusion [33].

In our study, both the axial- and the tangential map-guided nomograms showed significant improvement in terms of UDVA, CDVA, and MRSE. In the axial group, UDVA and CDVA improved from 0.12 ± 0.04 and 0.24 ± 0.08 to 0.28 ± 0.08 and 0.4 ± 0.1, respectively. Similarly, MRSE substantially decreased from −6.7 ± 3.3 to −1.2 ± 1.1*D* at 12 months after the procedure. In the tangential group, UDVA and CDVA improved from 0.09 ± 0.06 and 0.2 ± 0.1 to 0.5 ± 0.2 and 0.7 ± 0.2, respectively. MRSE substantially decreased from −4.9 ± 1*D* to −1.00 ± 1.6*D* at 12 months after the procedure. The tangential map-guided group had statistically significant better UDVA and CDVA, either in terms of absolute values or change values (Δ). While for MRSE, the change values (Δ), not the absolute ones, were significantly lower in the tangential group.

In a recent study, 3 types of rings were compared: 2 symmetric 160° ring segments, 320° rings and MyoRing. The authors reported their own nomograms for central cones defined on posterior elevation maps. MyoRing and 320° rings achieved better visual and topographic outcomes than symmetric ring segments. Trying to reflect this in our study, 22 out of 28 eyes (78.6%) in the tangential group compared to 12 out of 36 (33.3%) in the axial group received the 320° rings. This can explain the better visual outcomes obtained in the tangential group [34].

The better outcomes in the tangential group could be attributed to the higher proportion of the central/paracentral cones with more 320° rings implanted. Literature review for prior 320° segments' studies yielded only four reports which will be highlighted chronologically. The first study was conducted in Egypt by Israel and her colleagues who reported the outcomes for 4 types of rings: 160, 210, 320, and 355 [35]. After that, Yousif and Said compared 3 types of rings: 2 symmetric 160° ring segments, 320° rings, and MyoRing [34]. Recently, 2 reports from Brazil evaluated the 320 rings alone without a comparison group [36, 37].

Visual outcomes in our study are comparable or superior to all prior studies. Israel reported improved UCVA and CDVA from 0.02 to 0.06 to 0.16 and 0.32 respectively, and a reduction of spherical error from −9.5 to −0.5*D* [35]. Yousif and Said reported better visual and topographic outcomes with MyoRing and 320° rings compared symmetric ring segments. For the 320 rings, UCVA and CDVA significantly improved from 1.6 and 0.33 LogMAR to 0.25 and 0.05 LogMAR at 6 months, respectively. Moreover, SE was significantly reduced from −5.87 to −1.5*D* at 6 months follow-up [34].

Not far from this, Rocha and his colleagues reported improved UCVA and CDVA from 1.36 and 0.51 (LogMAR) to 0.63 and 0.18 (LogMAR), respectively, after 6 months of 320 rings' implantation [36, 37]. Including 128 eyes, Torquetti et al. conducted the largest study on the 320 rings. At 6 months of follow-up, mean CDVA significantly improved from 0.2 to 0.5, while SE was significantly reduced from −7.02 to −3.2*D*. However, one limitation to consider is using two techniques for ring implantation: manual and femto-assisted. Authors did not report a subgroup analysis for the technique which may confound the obtained outcomes [36].

In the axial group, *K*_max_ was significantly reduced from 53.6 ± 2.5*D* preoperatively to 48.8 ± 3.9*D* at 12 months, while in the tangential group, it was reduced from 54 ± 6.4*D* preoperatively to 50 ± 4.7*D* at 12 months. Similarly, average *K* reading (*K*_m_) showed significant reduction in both groups. In the axial group, it was significantly reduced from 51.7 ± 5.8*D* preoperatively to 45 ± 3.7*D* 12 months, while in the tangential group, it was reduced from 51.09 ± 1.4*D* preoperatively to 44.5 ± 2.7*D* at 12 months postoperatively.

This is in agreement with El-Raggal, who reported a statistically significant reduction in the mean keratometric reading from the preoperative values [38]. Similarly Shabayek and Alió reported that ICRS significantly decreased the keratometric values (*K*_max_ and *K*_m_) and significantly enhanced both the UDVA and CDVA as well as improving the corneal topography [39]. Also, Ibrahim et al., observed an approximate reduction of 3*D* in *K*_m_ [40].

It is not surprising to obtain better outcomes without providing an explanation for this. Such conflicting ambiguous reports, along with ours, raise many questions such as how do corneal rings really work and what does really count with them? We are not in the position to declare a nomogram to be followed by all surgeons. However, the discrepancy in the outcomes we present are too significant to discard. Considering the posterior corneal surface and high-order aberrations in further studies with larger sample size and long-term follow-up may elucidate the outcomes' influencer. Reaching a consensus for the terminology, including the cone location, and the nomograms for ICRS implantation is essential for a reproducible practice.

## 5. Conclusion

Both axial- and tangential map-based nomograms for ICRS significantly enhanced the visual and the topographic outcomes in keratoconus, with a “centralization tendency” for cone location by the tangential map. In addition, the tangential map-based nomogram yielded better visual and refractive outcomes especially in the peripheral cones. Larger studies to validate the tangential map-based nomogram along with other available nomograms are essential for a consensus in ICRS practice.

## Figures and Tables

**Table 1:** Baseline characteristics of study population.

Variable	Axial group (*n* = 36)	Tangential group (*n* = 28)	*P* value
Age	27.6 ± 3.8	23.7 ± 3.5	0.07
Gender			
Males	24 (67.7%)	12 (42.8%)	0.9
Females	12 (33.3%)	16 (57.2%)	
UDVA	0.12 ± 0.0.4	0.09 ± 0.06	0.2
CDVA	0. 24 ± 0.08	0.35 ± 0.1	0.4
MRSE (D)	−4.9 ± 1.00	−6.7 ± 3.3	0.1
*K*_m_ (D)	51.7 ± 5.8	51.09 ± 2.38	0.2
*K*_max_ (D)	53.6 ± 2.5	54 ± 6.4	0.9
*Q* value	−1.28 ± 0.53	−1.38 ± 1.30	0.7
Thinnest location (*µ*m)	395.3 ± 33.8	398 ± 29.7	0.2

**Table 2:** Cone location matching between both maps.

		Tangential map (*N* = 64)			Total	Matching rate (%)	*P* value
		Central	Paracentral	Peripheral			
Axial map (*N* = 64)	Central	4	2	0	6	67.6	0.01^*∗*+^
	Paracentral	4	10	2	16	62.5	
	Peripheral	0	26	16	42	38.1	
	Total	8	38	18	64		

**Table 3:** Type of ICRS implanted in both groups.

Type of ICRS arc/thickness	Axial group (*N* = 36) %	Tangential group (*N* = 28) %	*P*
320°/300	6 (16.7)	12 (42.9)	>0.01^*∗*^
320°/250	6 (16.7)	10 (35.7)	
160°/250	14 (38.9)	4 (14.3)	
160°/300	10 (27.7)	2 (7.1)	

**Table 4:** Alternative scenario for cone asymmetry based on Kera manufacturer's nomogram. Note: A, B, and C point the nomogram to follow according to manufacturer's asymmetry classification for cones. Nomogram A when the cone is to one side or 20 : 80, nomogram B for 40 : 60 cones, while nomogram C for 50 : 50 cones.

		Tangential map (*N* = 64)			Total	Matching rate (%)	*P* value
		A	B	C			
Axial map (*N* = 64)	A	4	24	4	32	12.5	0.01^*∗*+^
	B	6	4	14	24	16.7	
	C	0	6	2	8	25	
	Total	10	34	20	64		

^+^Fisher's exact test.

**Table 5:** Postoperative outcomes of both groups at follow-up visits.

Variable	3 months			6 months			12 months		
	Axial	Tangential	*P*	Axial	Tangential	*P*	Axial	Tangential	*P*
UDVA	0.23 ± 0.07	0.3 ± 0.07	0.04^*∗*^	0.25 ± 0.07	0.4 ± 0.1	0.02^*∗*^	0.28 ± 0.08	0.5 ± 0.2	0.01^*∗*^
CDVA	0.36 ± 0.1	0.35 ± 0.1	0.3	0.38 ± 0.1	0.6 ± 0.2	0.005^*∗*^	0.4 ± 0.1	0.7 ± 0.2	0.006^*∗*^
MRSE	−1.5 ± 1.8	−2.2 ± 1.5	0.5	−1.3 ± 1.7	−1.4 ± 1.2	0.9	−1.00 ± 1.6	−1.2 ± 1.1	0.8
*K*_max_	47.3 ± 2.4	45.5 ± 3.2	0.4	45.2 ± 3.3	44.9 ± 2.3	0.6	45 ± 3.7	44.5 ± 2.7	0.3
*K*_m_	50.3 ± 3.1	52 ± 4.8	0.5	49.7 ± 3.8	49 ± 4.6	0.3	48.8 ± 3.9	50 ± 4.7	0.6
*Q* value	−0.6 ± 0.3	−0.5 ± 0.7	0.3	−0.45 ± 0.8	−0.39 ± 0.69	0.2	−0.4 ± 0.6	−0.38 ± 0.5	0.5
Thinnest location	451 ± 49.1	409 ± 35.8	0.1	455 ± 21.2	443 ± 36.7	0.2	467 ± 15.2	428 ± 39.9	0.4

**Table 6:** Value of change in outcomes of both groups at follow-up visits.

Variable	3 months			6 months			12 months		
	Axial	Tangential	*P*	Axial	Tangential	*P*	Axial	Tangential	*P*
Δ UDVA	0.11 ± 0.07	0.2 ± 0.09	0.02^*∗*^	0.13 ± 0.08	0.3 ± 0.2	0.03^*∗*^	0.15 ± 0.1	0.35 ± 0.2	0.01^*∗*^
Δ CDVA	0.12 ± 0.07	0.1 ± 0.19	0.8	0.13 ± 0.04	0.25 ± 0. 2	0.002^*∗*^	0.15 ± 0.05	0.4 ± 0.1	0.003^*∗*^
Δ MRSE	3.3 ± 1.3	4.5 ± 2.9	0.3	3.7 ± 1.2	5.3 ± 3.1	0.04^*∗*^	3.9 ± 1.1	5.4 ± 3.1	0.03^*∗*^
Δ *K*_max_	−4.5 ± 1.3	−4.7 ± 2.3	0.2	−5.4 ± 2.09	−5.98 ± 1.69	0.2	−5.8 ± 0.8	−6.2 ± 3.1	0.3
Δ *K*_m_	−3.3 ± 1.2	−4.00 ± 1.8	0.5	−3.6 ± 1.3	−4.2 ± 2.9	0.7	−4.7 ± 1.2	−6.00 ± 1.2	0.1
Δ *Q* value	−0.45 ± 0.2	−0.5 ± 0.3	0.8	−0.4 ± 0.5	−0.6 ± 1.5	0.5	−0.5 ± 0.6	−0.65 ± 0.3	0.4
Δ thinnest location	32.6 ± 16.6	20.5 ± 12.2	0.3	7.3 ± 5.4	19.2 ± 14.7	0.4	25.1 ± 14.1	26.1 ± 12.7	0.9

**Table 7:** Value of change in outcomes of both groups at follow-up visits for cases with cone location discrepancy between maps. ThL: thinnest corneal location.

	3 months			6 months			12 months		
	Axial (*n* = 18)	Tangential (*n* = 14)	*P*	Axial (*n* = 18)	Tangential (*n* = 14)	*P*	Axial (*n* = 18)	Tangential (*n* = 14)	*P*
Δ UDVA	0.2 ± 0.1	0.3 ± 0.08	0.03^*∗*^	0.25 ± 0.1	0.35 ± 0.2	0.03^*∗*^	0.15 ± 0.1	0.45 ± 0.1	0.02^*∗*^
Δ CDVA	0.17 ± 0.07	0.19 ± 0.1	0.7	0.13 ± 0.07	0.3 ± 0. 2	0.002^*∗*^	0.15 ± 0.05	0.45 ± 0.1	0.002^*∗*^
Δ MRSE	3.7 ± .3	6.5 ± 2.9	0.4	3.8 ± 1.2	7.2 ± 2.1	0.03^*∗*^	3.9 ± 0.9	8.4 ± 2.7	0.03^*∗*^
Δ *K*_max_	−4.3 ± 1.3	−7.2 ± 2.3	0.2	−5.1 ± 2.09	−7.8 ± 1.69	0.3	−5.5 ± 0.8	−8.3 ± 2.1	0.04^*∗*^
Δ *K*_m_	−4.2 ± 1.2	−4.7 ± 1.8	0.5	−3.8 ± 1.3	−4.2 ± 2.9	0.7	−4.7 ± 1.2	−6.00 ± 1.5	0.1
Δ *Q*	−0.4 ± 0.2	−0.42 ± 0.3	0.6	−0.3 ± 0.2	−0.52 ± 0.3	0.5	−0.3 ± 0.6	−0.6 ± 0.4	0.4
Δ ThL	31.6 ± 13.6	30.5 ± 9.2	0.3	10.1 ± 2.3	22.4 ± 12.7	0.4	23.1 ± 11.2	28.3 ± 10.3	0.9

**Table 8:** The value of change at 12 months for both groups with different cone locations. ThL: thinnest corneal location.

	Central cones			Paracentral			Peripheral		
	Axial (*n* = 2)	Tangential (*n* = 4)	*P*	Axial (*n* = 8)	Tangential (*n* = 18)	*P*	Axial (*n* = 24)	Tangential (*n* = 6)	*P*
Δ UDVA	0.2 ± 0.1	0.3 ± 0.1	0.2	0.23 ± 0.1	0.35 ± 0.2	0.5	0.15 ± 0.1	0.4 ± 0.1	0.001^*∗*^
Δ CDVA	0.2 ± 0.08	0.3 ± 0.05	0.5	0.25 ± 0.07	0.4 ± 0.1	0.3	0.1 ± 0.1	0.45 ± 0.05	0.002^*∗*^
Δ MRSE	4.2 ± .1	5.1 ± 1.6	0.6	4.6 ± 1.4	5.6 ± 2.3	0.4	3.3 ± 0.9	5.3 ± 2.7	0.01^*∗*^
Δ *K*_max_	−5.1 ± 1.3	−6.3 ± 2.3	0.3	−5.5 ± 2.09	−5.9 ± 1.69	0.4	−6.3 ± 0.8	−6.5 ± 1.1	0.06
Δ *K*_m_	−4.5 ± 1.2	−5.8 ± 1.4	0.3	−4.6 ± 1.3	−6.7 ± 1.8	0.2	−4.9 ± 2.5	−5.2 ± 1.5	0.1
Δ *Q*	−0.4 ± 0.2	−0.6 ± 0.2	0.4	−0.5 ± 0.2	−0.7 ± 0.4	0.5	−0.5 ± 0.6	−0.5 ± 0.3	0.5
Δ ThL	26.3 ± 1.5	22.4 ± 9.2	0.2	24.8 ± 2.5	27 ± 12.7	0.3	27.2 ± 11.2	26.1 ± 10.3	0.7

## Data Availability

The data used to support the findings of this study are included within the article.

## Supplementary Materials

Supplementary Materials Keraring intrastromal corneal ring segments nomogram.
