# Supplementary figures and images for: Evaluation of a Tangential Map-Based Nomogram for Intrastromal Corneal Ring Segments' Implantation in Keratoconus: One Year Results
Source: J Ophthalmol. 2020 Feb 19;2020:3983508. doi: 10.1155/2020/3983508 (PMC7049859; doi:10.1155/2020/3983508)

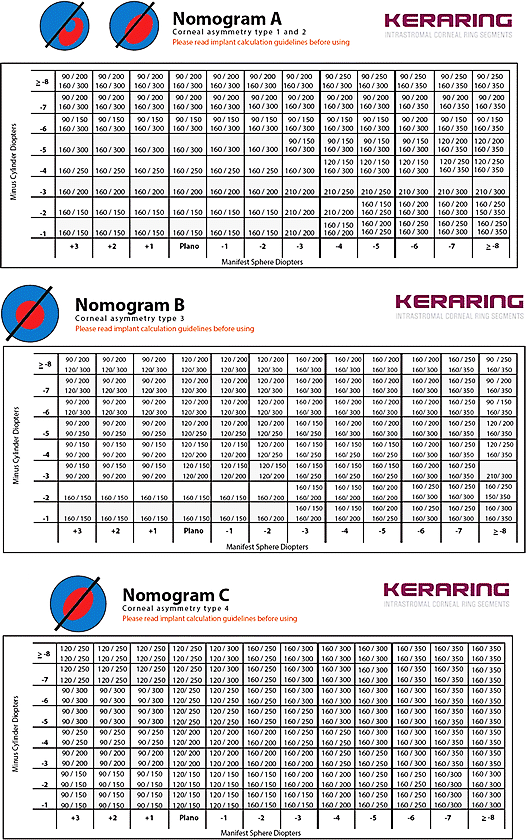

Supplement: Supplementary Materials — Keraring intrastromal corneal ring segments nomogram. [file 3983508.f1.tif]
